# Effect of hyperparathyroidism on coagulation: a global assessment by modified rotation thromboelastogram (ROTEM)

**DOI:** 10.3906/sag-2012-247

**Published:** 2021-05-06

**Authors:** Göknur YORULMAZ, Ahmet Toygar KALKAN, Aysen AKALIN, Elif Sevil ALAGÜNEY, Eren GÜNDÜZ, Bartu BADAK, Betül AYDIN BUYRUK, Nur KEBAPÇI, Belgin EFE, Muzaffer BİLGİN, Olga Meltem AKAY

**Affiliations:** 1 Department of Endocrinology and Metabolism, Faculty of Medicine, Eskişehir Osmangazi University, Eskişehir, Turkey; 2 Department of Hematology, Faculty of Medicine, Eskişehir Osmangazi University, Eskişehir, Turkey; 3 Departments of General Surgery, Faculty of Medicine, Eskişehir Osmangazi University, Eskişehir, Turkey; 4 Department of Biostatistics and Medical Informatics, Faculty of Medicine, Eskişehir Osmangazi University, Eskişehir, Turkey; 5 Department of Hematology, Faculty of Medicine, Koç University, Istanbul

**Keywords:** Hyperparathyroidism, coagulation, thromboelastogram, ROTEM

## Abstract

**Background/aim:**

Hyperparathyroidism is an endocrine disorder characterized by hypercalcemia. Because of calcium’s effects on parathyroid glands, bone, intestines, and kidneys, it has an important place in homeostasis. The results of studies regarding hyperparathyroidism hemostasis are conflicting. Thromboelastography helps to evaluate all steps of hemostatic system. Our aim in this study was to investigate the possible role of hemostatic mechanisms in the development of thrombosis in hyperparathyroid patients with the modified rotation thromboelastogram (ROTEM).

**Materials and methods:**

Twenty-two patients with primary hyperparathyroidism (PHPT) and 20 healthy controls were involved. This study was conducted in Eskisehir Osmangazi University Faculty of Medicine, Endocrinology and Hematology clinics for 2 years. The complete blood count, fibrinogen, D-dimer levels, prothrombin time, activated prothrombin time, and ROTEM parameters [clot formation time (CFT), clotting time (CT), and maximum clot formation (MCF)] were determined by two activated tests, INTEM and EXTEM analyses. A thromboelastographic evaluation was performed in the preoperative and postoperative (3 months after surgery) periods.

**Results:**

In INTEM assay, the CT (p = 0.012) and CFT (p = 0.07) values were increased in preoperative PHPT patients compared with the control group. Although there was a decrease in the postoperative CT and CFT values, no statistical difference was found.

**Conclusion:**

The prolongation of the CT and CFT values were consistent with a hypocoagulable state in patients with PHPT. Hyperparathyroidism causes a hypocoagulable state that can be successfully assessed by ROTEM. Hemostatic changes, do not seem to have an effect on increased cardiovascular mortality.

## 1. Introduction

Calcium ion plays an important role in human physiology such as cardiac automaticity, skeletal muscle and smooth muscle contraction and relaxation, blood coagulation, neuronal transmission, synaptic transmission, hormone secretion, mitotic separation, cilial movement, bone metabolism, and neurotransmitter release. Hypercalcemia is a metabolic disease with a clinical spectrum ranging from asymptomatic biochemical abnormalities to life-threatening disorders. Parathormone is the main regulator of extracellular calcium concentration [1,2]. Primary hyperparathyroidism (PHPT) increases calcium levels and presents with abnormalities in both coagulation and fibrinolysis. The literature contains studies about the relationship between hyperparathyroidism and thrombosis, which reveal hypercoagulability and hypofibrinogenemia [3–7]. In addition to high calcium status, a tendency to coagulation is shown in secondary hyperparathyroidism [8]. Studies evaluating coagulation disorders in patients with primary hyperparathyroidism are limited, and it has not yet been fully assessed whether their effects on coagulation are due to hypercalcemia or primary hyperparathyroidism. The results of a limited number of studies do not always support each other [3–8]. Hypercalcemia may cause thrombosis due to vascular smooth muscle contraction and vasoconstriction or platelet aggregation and coagulation factors. Additionally, hypercalcemia may effect renal water and sodium excretion resulting with dehydration. Considering all these different mechanisms, the mechanism of thrombosis in primary hyperparathyroidism is still unclear [9–12].

Thromboelastography (TEG) is used to evaluate the components of all steps in hemostasis [13]. Rotational thromboelastogram (ROTEM), which developed from the TEG technology, is superior to TEG even it has a good correlation with the conventional method. It was possible to evaluate heparin effect in differential diagnosis, platelet and fibrinogen contribution in clot strengthening, and diagnosis of hyperfibrinolysis with the addition of activators or inhibitors in ROTEM, as a modified TEG method. TEG has been used to explain various clinical cases related to hypercoagulation such as postoperative hypercoagulation and ischemic heart disease. Coagulation disorders which cannot be detected in routine tests may be noticed by ROTEM. ROTEM analysis has already placed in diagnostic and treatment algorythms for patients with bleeding. Also, ROTEM may be used to measure hypercoagulopathy in several situations [13].

In this study, we aimed to investigate if the hemostatic system plays a role in the development of thrombosis in patients with PHPT. We evaluated coagulation status of patients with PHPT to determine whether they could be diagnosed based on the prolongation of clotting time (CT) and clot formation time (CFT) values in both INTEM and EXTEM assays. In this study, it was aimed to compare the homeostatic system of PHPT cases with the preoperative and postoperative control group, as TEG is a valuable tool for clinicians to diagnose coagulopathy early.

## 2. Materials and methods

Twenty-two (20 females, two male) newly diagnosed symptomatic PHPT patients (age: 55 ± 11 years) and 20 age-matched healthy normocalcemic female controls (age: 49 ± 7 years) were included in this cohort study. Patients that presented to the endocrinology outpatient clinic for PHPT between January 2018–December 2019 were included in the study. The patients were evaluated before and at the third month after parathyroidectomy. Patients with known hematological or coagulation diseases, using anticoagulant agents, and patients with liver or renal failure were not included in this study.

The participants did not have any additional cardiovascular risk factor. The diagnosis of PHPT was based on high levels of albumin-corrected calcium, high parathormone intact, and hypercalciuria. Before the surgical procedure, preoperative imaging studies (neck ultrasonography and dual-phase technetium-99m sestamibi scintigraphy) were performed for localization. The pathology results of all operated patients were consistent with parathyroid adenomas. None of the individuals were receiving any antiaggregant or anticoagulant therapy. Samples were collected from all patients to analyze the complete blood count (CBC), prothrombin time (PT), activated prothrombin time (aPTT), D-dimer, and TEG. Written informed consent was obtained from all individuals participating in the study. Control group was composed of voluntary individuals. The study was approved by the local ethic committee of the university.

### 2.1. Sample collection

Blood was collected from all participants under minimum stasis from antecubital peripheric veins. Blood samples were drawn into EDTA tubes (Becton Dickinson, Plymouth, UK) for CBC and 4.5-mL vacutainers (Becton Dickinson, Plymouth, UK) containing 3.2% trisodium citrate with a citrate/blood ratio of 1:9 for TEG and coagulation profile. Both control group and patient group samples were taken in morning fasting. CBC and coagulation analyses were undertaken using an automated hematology analyzer, ADVIA 2120i (Siemens, New York, USA). Conventional coagulation parameters, namely PT and aPTT, fibrinogen, and D-dimer were obtained using an automatic coagulation analyzer (BCS/XP, Siemens Healthcare Diagnostics, Malburg, Germany). The normal ranges for these tests used in our laboratory are 8.2–13.2 s for PT, 24–40 s for aPTT, 200–400 mg/dL for fibrinogen, and 0.00–0.50 mg/L for D-dimer. Thromboelastographic analyses were carried out using the ROTEM Coagulation Analyzer (Pentapharm, Munich, Germany). Three hundred μL of citrated whole blood, which was recalcified with 20 μL 0.2mol/LCaCl2 (star-TEM; Pentapharm, Munich, Germany) was used for each test. Intrinsic rotational thromboelastography (INTEM) and extrinsic rotational thromboelastography (EXTEM) tests were performed on each sample.

All the samples were analyzed within 30–90 min of blood collection. CT, CFT, and MCF (maximal clot firming) data were analysed to determine the pattern of clot formation. CT measures the time from start of measurement until initiation of clotting, CFT is the time from initiation of clotting until a clot firmness of 20 mm is detected and MCF describes the maximum amplitude, indicative of the firmness of the clot.

### 2.2. Statistical analysis

Continuous data are given as mean ± standard deviation. Categorical data are given as percentage (%). Shapiro–Wilk’s test was used to investigate the compatibility of the data for normal distribution. When comparing normally distributed groups, independent sample t-test analysis was used for cases with two groups. In comparison of groups that do not conform to normal distribution, the Mann–Whitney U test was used for cases where the number of groups was two. In comparing the values at different measurement times, Wilcoxon test was used when the number of groups was two. IBM SPSS Statistics 21.0 (IBM Corp. Released 2012. IBM SPSS Statistics for Windows, v. 21.0. Armonk, NY: IBM Corp.) program was used in the application of the analyzes. For statistical significance, a value of p < 0.05 was accepted as the criterion.

## 3. Results

Twenty-two (20 female, 2 male) newly diagnosed symptomatic PHPT patients (age: 55 ± 11 years) and 20 age-matched healthy normocalcemic female controls (age: 49 ± 7 years) were included in the study. The clinical characteristics of the PHPT patients are summarized in Table 1. The parathormone, alkaline phosphatase and calcium levels were decreased postoperatively, as expected. As expected, the parathormone, alkaline phosphatase, and calcium levels improved in the postoperative period (Table 1).

The PT, PTT, fibrinogen, and D-dimer levels did not show any difference preoperatively and postoperatively (Table 2). The correlation of the thromboelastogram results with the PT, PTT, fibrinogen, and D-dimer levels of the patients were not statistically significant. There was no correlation between the EXTEM and INTEM parameters and the platelet count, parathormone, D-dimer, calcium, and fibrinogen levels. The preoperative CT and CFT were prolonged in the hyperparathyroid patients according to the EXTEM and INTEM analyses (p < 0.05). However, the MCF levels did not show any significant difference preoperatively (Table 3). The preoperative and postoperative CT, CFT, and MCF parameters of the hyperparathyroid patients obtained from INTEM and EXTEM did not show any significant difference (Table 4).

## 4. Discussion

The most important results of this research was that the global clotting process was determined by ROTEM, and the CT and CFT values were observed to be prolonged in PHPT. TEG measures the viscoelastic properties of blood and gives information about all steps of the coagulation and fibrinolytic processes such as, plasma factors, platelets, and leukocytes of all stages of the coagulation and fibrinolytic processes [13]. The CT and CFT values of the patients were still elevated at three months after the operation. CT and CFT are affected by the activities of coagulation factors, and the prolongation of the CT and CFT values in the present study confirmed the presence of a hypocoagulable state. While other studies have shown a tendency of patients toward a prothrombotic status [3], in this study consistent with a hypocoagulable state is obtained. In view of these findings, it is considered that the increased cardiovascular mortality in patients with PHPT may be associated with traditional cardiovascular risk factors rather than hemostatic changes. Increased risk of cardiovascular mortality in PHPT is known and does not improve after parathyroidectomy [14–16]. The prevalence of traditional cardiovascular risk factors is increased in patients with PHPT [17]. Nevertheless, the results about the effects of PHPT on the coagulation-fibrinolytic system in the published data is conflicting. Erem et al. evaluated the hemostatic system in PHPT and showed an increase in some of the prothrombotic factors [3,4]. In another study, Chertok-Shacham et al. reported a positive relationship between the plasma PAI-1 antigen and parathormone levels in 35 patients symptomatic PHPT who do not have any evidence of cardiovascular disease [18]. Boas et al. evaluated patients with chronic kidney failure and secondary hyperparathyroidism scheduled for total parathyroidectomy and reported that they exhibited pro-thrombotic TEG parameters. In these studies, the researchers attempted to evaluate the coagulation cascade with various factors in different steps. The inconsistency may result from different methods used to exclude the effect of concomittant cardiovascular risk factors and also differences of serum calcium levels. All studies concluded with a proposal of studies with more patients, on the coagulation-fibrinolytic function of patients with PHPT. There are ongoing studies investigating the effect of serum calcium levels on platelet aggregation, coagulation, and TEG parameters in healthy people [8,19]. Recent study investigated the platelet functions of patients with hyperparathyroidism using platelet aggregation tests and determined that neither primary and secondary hyperparathyroidism nor serum calcium levels significantly affected platelet functions [20]. Although Elbers et al. studied different groups of hyperparathyroidism, they did not determine any effect of hyperparathyroidism on coagulation or fibrinolysis [21]. In the current study, the MCF values, which are affected by platelet count and function, did not show any significant difference in the PHPT patients compared to the controls. The difference in MCF was also nonsignificant between the preoperative and postoperative measurements of the patient group. The ROTEM-MCF data obtained from this study confirm the idea that PHPT does not affect platelets.

PHPT is the most common cause of postmenopausal hypercalcemia. Studies show that the frequency of PHPT had a F/M ratio of 3 to 4:1 [22–24]. The majority of our patient group consisted of postmenopausal female patients and we only had two male cases. Therefore, we chose to form the control group with female patients. The small number of patients, especially small number of male patients is a limitation of this study. Another limitation of the study is the lack of asymptomatic PHPT group.

In conclusion, hyperparathyroidism causes a hypocoagulable state that can be successfully assessed by ROTEM. Rather than hemostatic changes, cardiovascular risk factors such as hypertension, glucose intolerance, and dyslipidemia seem to be responsible for an increased risk of cardiovascular mortality. Principally, contrary to popular belief, since at least one group of PHPT patients has an increased bleeding tendency, they should also be followed up in this way in daily life and preoperatively. However, further studies in greater series are needed to elucidate the hemostatic system in patients with PHPT.

## Figures and Tables

**Table 1:** Clinical parameters of hyperparathyroid patients.

	Preoperative (n = 22)	Postoperative (n = 22)	P

Parathormone (pg/mL)	344 ± 225.7	43.69 ± 14.5	0.001¥¥
	229.1 (152.5–462.5)	43.85 (34.40–48.06)	

Vitamin D	19.94 ± 16.01	22.98 ± 10.11	0.216¥¥
	14.77 (10.3–23.00)	24.5 (15.46–26.50)	

Calcium (mg/dL)	11.9 ± 1.3	9.2 ± 0.37	<0.001¥
	13.5 (12.73–14.65)	9.28 (9.01–9.60)	

Phosphorus (mg/dL)	2.1 ± 0.5	3.5 ± 0.4	<0.001¥
	2.07 (1.82–2.55)	3.38 (3.14–3.71)	

Alkaline phosphatase (U/L)	144.3 ± 87.9	94.1 ± 47.3	0.006¥¥
	155.6 (120.80–208.60)	103.30 (62.60–146.50)	

Hemoglobin (mg/dL)	13.48 ± 1.55	13.17 ± 2.05	0.130¥
	12.67 (11.96–14.09)	12.80 (11.43–15.16)	

Leucocyte count (10^3^/μL)	7.838 ± 2.575	7.048 ± 3.476	0.204¥¥
	7.50 (6.50–8.50)	6.50 (5.50–7.25)	

Platelet count (10^3^/μL)	298.3 ± 26.6	252.1 ± 68.6	0.454¥
	231.50 (194.750–283.75)	243.50 (208.50–300.75)	

Glucose (mg/dL)	92.1 ± 9.5	91.1 ± 9.3	0.527¥
	91.00 (85.50–98.00 )	90.00 (86.00–95.00)	

* SD: standard deviation,

¥ Paired sample t test,

¥¥ Wilcoxon test.

The values are presented as mean+/− SD and median (Q1–Q3).

**Table 2:** Comparison of the PT, aPTT, INR, PLT counts of the hyperparathyroid patients (preoperative measurements) and healthy controls.

	Preoperative (n = 22)	Postoperative (n = 22)	Preoperative/Postoperative P	Control (n = 20)	P

Platelet count (103/μL) (mean ± SD)	298.3 ± 26.6	252.1 ± 68.6	0.88^¥^	224.2 ± 40.2	Pre-C: <0.001¥¥ Post-C: 0.1204¥¥
	231.50 (194.750 – 283.75)	243.50 (208.50–300.75)		222.0 (186.00–285.00)	

PT (sec.) *	11.7 ± 0.9	11.9 ± 1	0.28^¥^	11.5 ± 0.6	Pre-C: 0.406¥¥ Post-C: 0.1286¥¥
	11.50 (11.15 – 12.40)	11.80 (11.20–12.75)		11.50 (11.30–11.85)	

INR**	1.0 ± 0.09	1.0 ± 0.1	0.42^¥^	1.0 ± 0.6	Pre-C: 1.000¥¥ Post-C: 1.000¥¥
	1.00 (0.90 – 1.07)	1.03 (1.00–1.05)		1.05 (0.67–1.46)	

aPTT (sec.) ***	28.6 ± 3.7	29.1 ± 4.4	0.84^¥^	27.7 ± 1.9	Pre-C: 0.334¥¥ Post-C: 0.1961¥¥
	28.04 (26.00–31.25)	28.00 (26.25–29.25)		27.66 (26.56–29.35)	

* PT, prothrombin time,

** INR, international normalization ratio, APTT, activated partial thromboplastin time,

*** ¥ Wilcoxon Test

¥¥ Mann–Whitney U test.

The values are presented as mean+/− SD and median (Q1–Q3). Preoperative vs. postoperative comparison was done with Wilcoxon signed rank test and patients (either preoperative or postoperative) vs. control comparison was done with Mann–Whitney U test.

**Table 3:** Comparison of the thromboelastometry parameters between the hyperparathyroid patients (preoperative measurements) and healthy controls.

	Patient group (preoperative) (n =22)	Control group (n = 20)	P¥

INTEM CT*	221.8 ± 69.46	179.9 ± 20.72	0.012
	223.50 (187.75–260.00)	183.00 (169.00–190.00)	

INTEM CFT**	145.0 ± 107.15	87.85 ± 16.78	0.007
	117.50 (90.50–156.50)	84.00 (74.50–100.50)	

INTEM MCF***	58.63 ± 6.88	60.85 ± 4.17	0.157
	59.00 (55.75–62.25)	62.00 (58.50 – 63.50)	

EXTEM CT	82.86 ± 17.81	76.28 ± 15.12	0.032
	85.50 (77.75–94.00)	79.00 (71.00–82.50)	

EXTEM CFT	144.04 ± 64.36	99.85 ± 23.17	0.003
	133.50 (107.25–166.50)	94.00 (80.00–121.50)	

EXTEM MCF	59.45 ± 12.70	62.76 ± 3.75	0.6
	63.00 (58.75–65.25)	63.00 (61.00–66.00)	

* CT: clotting time,

** CFT: clot formation time,

*** MCF: maximal clot firmness,

¥ Mann–Whitney U test.

**Table 4:** Comparison of the preoperative and postoperative thromboelastometry parameters of the hyperparathyroid patients.

	Preoperative measurement (n = 2)	Postoperative measurement (n: 22)	P¥

INTEM CT*	221.8 ± 69.46	221.45 ± 72.11	0.984
	223.50 (187.75–260.00)	183.00 (169.00–190.00)	

INTEM CFT**	145.0 ± 107.15	107.90 ± 51.24	0.181
	117.50 (90.50–156.50)	84.00 (74.50–100.50)	

INTEM MCF***	58.63 ± 6.88	61.20 ± 5.44	0.062
	59.00 (55.75–62.25)	62.00 (58.50–63.50)	

EXTEM CT	82.86 ± 17.81	88.40 ± 39.73	0.553
	85.50 (77.75–94.00)	79.00 (71.00–82.50)	

EXTEM CFT	144.04 ± 64.36	134.04 ± 55.92	0.596
	133.50 (107.25–166.50)	94.00 (80.00–121.50)	

EXTEM MCF	59.45 ± 12.70	65.4 ± 6.96	0.080
	63.00 (58.75–65.25)	63.00 (61.00–66.00)	

* CT: clotting time,

** CFT: clot formation time,

*** MCF: maximal clot firmness

¥ Wilcoxon test.

The values are presented as mean+/− SD and median (Q1–Q3).
